# Research note: top-view walking features as proxy for walking ability in broilers and their potential use in breeding

**DOI:** 10.1016/j.psj.2025.106215

**Published:** 2025-12-09

**Authors:** Malou van der Sluis, Marjaneh Taghavi, István Fodor, Elianne G.T. van der Valk, Jesus Arango, Esther D. Ellen

**Affiliations:** a Animal Breeding and Genomics, Wageningen University & Research, 6700 AH Wageningen, Netherlands; b Research and Development, Cobb Vantress LLC, Arkansas 72761, USA

**Keywords:** gait score, automation, computer vision, chickens, genetics

## Abstract

Impaired walking ability is a welfare concern that affects many broilers. Walking ability can potentially be improved through genetic selection, but this requires objective data on large numbers of broilers in the breeding program. Here, it was studied whether top-view video recordings of broilers in a walkway can provide insight into walking ability. Five walking features were analysed from the videos for 595 female, pure-line broilers: 1) lateral body oscillation (**LBO**), 2) number of steps taken (**STEP**), 3) mean overall speed (**MEAN_SPEED**), 4) maximum forward moving speed (**MAX_SPEED**), and 5) maximum forward acceleration (**MAX_ACC**). Birds’ gait scores (**GS**) were furthermore manually assessed (*n* = 153 with GS1; *n* = 344 with GS2; *n* = 87 with GS3 and *n* = 11 with GS4) and the heritabilities of the manual GS and the top-view derived walking features were estimated. The walking features all differed between GS groups, with a higher level of LBO in GS2 birds than in all other GS groups and a decrease in MEAN_SPEED and MAX_SPEED with increasing GS from GS1 to GS3. MEAN_ACC was lower for GS3 than GS1 birds. An interaction effect between body weight and GS was furthermore observed, in which - within the different body weight levels - higher GS levels were associated with more steps taken. Moreover, LBO, STEP, MEAN_SPEED and manual GS were found to be low to moderately heritable in broilers. Overall, the results of this study indicate that top-view derived features can provide insight into broiler walking ability and these outcomes can contribute to breeding for improved walking ability in broilers.

## Introduction

There is potential to genetically select broilers for improved walking ability (Kapell et al., 2025). However, to be able to improve walking ability through breeding, data need to be collected on large numbers of broilers in the breeding program. This is challenging, as walking ability is commonly scored manually, which is time-consuming, and interrater agreement for manually determined gait scores (**GS**) is relatively low, even for trained observers (Fodor et al., 2023).

Automated approaches for recording walking ability could contribute to making gait scoring faster and more objective. In earlier work (Fodor et al., 2024), it was studied whether top-view video recordings of broilers in a walkway could provide insight into broiler walking ability. It was shown that birds with poorer gait (GS3+) had longer completion times, higher step counts and a trend for higher lateral body oscillation levels in the walkway setup. Using these walking features, birds with GS3+ could be distinguished from birds with a lower GS, but distinguishing between birds with GS1 and GS2 was challenging.

In this study, we collected similar video data on a larger group of pure-bred broilers to evaluate whether additional top-view walking features can better distinguish broilers with good to moderate walking ability. We also estimated the heritability of manual GS and the top-view-derived walking features. The results of this study provide more insight into top-view-derived proxies for GS and contribute to breeding for improved walking ability in broilers.

## Materials and methods

### Ethical statement

Data were collected at a Cobb research farm, under control of Cobb. This study is not considered to be an animal experiment under the Law on Animal Experiments, as confirmed by the Dutch Animal Welfare Body (July 11, 2022, Lelystad, the Netherlands).

### Animals and housing

A total of 1,377 birds were housed in a pen of 5.7 × 19 m (108.3 m^2^), with 178 drinker nipples and 21 feed pans available, and wood shavings as bedding. The birds received an IB vaccination in the hatchery at day of hatch. The birds were kept under a 23L:1D light schedule from 1 to 7 days of age, and a 18L:6D schedule from 8 days of age onwards. Birds originated from one pure-bred line. In total, 53 sires and 427 dams of varying age were used as parents. Each sire was mated with approximately 8 dams, resulting in 3.2 offspring per dam.

### Data collection

Video data were collected from 1,377 birds aged 32–33 days walking through a specially designed walkway across two days: mainly males on day one and mainly females on day two. For this study, we analyzed the 33-day-old females recorded on day two (*n* = 630). The walkway was located inside the home pen and consisted of two parallel corridors (each 2.8 × 0.4 × 0.75 m; length x width x height) with wood shavings on the floor and wooden walls to prevent birds from seeing those in the adjacent corridor.

In these corridors, birds were filmed from the front, back and two locations from the top (i.e., four cameras per corridor) using Reolink RLC-510A cameras (Reolink, Hong Kong, China). These cameras recorded continuously, with a framerate of 25 frames per second and a resolution of 1920 × 2560 pixels. We here used the recordings from the top-view cameras near the end of the walkway, that were fixed at a height of approximately 1.8 m above the walkway floor.

Before entering the walkway, birds were individually handled to record sex, body weight (**BW**), foot pad dermatitis, hock burn, and leg abnormalities (bowed/rotated), although only sex and BW were used in this study. Each bird was then placed at the start of one of the two corridors and allowed to walk to the end, where it was collected and returned to its home pen. Birds that did not walk were gently nudged using a wire mesh panel on a stick, without a fixed nudging protocol. During walking, GS were manually assessed by one of two experienced observers (one per corridor) following van der Sluis et al. (2021). This scoring system ranged from 0 to 5, with a score of 0 representing birds that walk very well and a score of 5 representing birds that can barely walk and use their wings for support when walking. There were no birds with GS0 in the dataset and birds with GS5 (*n* = 2) were excluded as they did not complete sufficiently long tracks in the walkway for further analysis (see further on).

### Video processing

The video processing was similar to the approach in Fodor et al. (2024). Before recording, camera calibration with a 6 × 6 checkerboard was performed to enable frame undistortion. During data collection, each bird’s start and end times in the walkway were noted, and frames from that interval were extracted at 25 fps. A pre-trained YOLOv8 pose model was adapted to detect broilers and their head keypoints. A total of 500 images were annotated using the Computer Vision Annotation Tool (CVAT; v1.1.0), with 200 training and 50 validation images selected from each walkway. Two models were trained for 200 epochs with a batch size of 2, using early stopping (patience of 50 epochs) to prevent overfitting. The best model, based on performance on the validation set, achieved an mAP50-95 (mean Average Precision) of 0.88 for bounding box detection and 0.99 for head keypoint detection for both walkways.

### Top-view features

Using the bounding box and head keypoint coordinates, the bounding box center was used as the bird’s position. Two filters were applied: (1) only forward-moving tracks were retained, allowing up to 5 pixels of apparent backward motion to account for pendulum-like gait; and (2) detections outside the walkway were removed. Gaps created by filtering were linearly interpolated if shorter than 5 frames (0.2 s), otherwise left missing. For each bird, the longest complete track was retained, and birds with tracks covering less than 50 % of the walkway were excluded. The remaining center point data were smoothed with a seven-frame moving average. Five top-view walking features were then derived per bird:

1. Lateral body oscillation (**LBO**): the bird’s overall movement trajectory was separated from its oscillatory deviations (as in Fodor et al. (2024)) by fitting a 5th-order polynomial to the track. LBO was quantified as the mean absolute residual from this polynomial.
2. Number of steps taken (**STEP**): the number of times the center point of the bounding box (and thus, bird) crossed the trajectory, corrected for the length of the walking trajectory of the bird.
3. Mean overall speed of movement (**MEAN_SPEED**): the length of the track (which could differ per bird, as described earlier) was divided by the time span used to cover that distance, to obtain a movement speed in pixels per second.
4. Maximum forward moving speed (**MAX_SPEED**): forward speed was calculated from the change in the bounding box center between consecutive frames, using only the x-direction (not the Euclidean distance). The maximum forward speed per bird (pixels per second) was taken as the MAX_SPEED.
5. Maximum forward acceleration (**MAX_ACC**): the change in velocity between consecutive frames was determined per bird, and the maximum value was kept as MAX_ACC.

After calculating these walking features per bird, extreme outliers were removed based on a threshold of three times the interquartile range. After all these quality control steps, a sample size of 595 broilers (94 % of the original sample size) remained.

### Statistical analyses of the walking features in relation to gait scores

All statistical analyses were performed in R version 4.4.1. To assess differences in body weight between the different GS groups (4 levels, GS1 to GS4), an ANOVA was implemented. The relationship of each of the walking features with walking ability was studied using linear models, in which the walkway was included as a fixed effect to account for a potential influence of the location of the walkway, as well as the associated gait scorer. The walking feature (LBO, STEP, MEAN_SPEED, MAX_SPEED or MAX_ACC, respectively) was initially modelled as a function of the GS group and the walkway as factors, BW as a covariate, and the two-way interaction between GS group and BW. Non-significant (*p* > 0.05) interactions were removed, and the models were subsequently refitted. Pairwise comparisons were performed using the emmeans package (version 1.10.3), and p-values were adjusted using Tukey contrasts. Moreover, to assess whether the walking features combined could provide insight into broiler gait, a Principal Component Analysis was performed, using only complete cases in the data (*n* = 548).

### Genetic analyses

R version 4.4.1 was used to decide which fixed effects to be included in the model to assess genetic parameters. For all five walking features and manually-determined GS, the model included only a fixed effect for walkway. The software package ASReml (version 4.2) was used to estimate genetic parameters. For each trait, the linear animal model was fitted according to Equation (1).

(1) y=Xb+Za+e

where y is a vector of the observed trait (LBO, STEP, MEAN_SPEED, MAX_SPEED, MAX_ACC, GS or BW), b is a vector of the fixed effect (walkway) with incidence matrix X linking observations to fixed effects, a is a vector of the breeding values, with incidence matrix Z linking observations on individuals to their breeding values, and e is a vector of random residuals. The variance structure of the model terms are $var\left[a\right]=A\sigma_{A}^{2}$ and $var\left[e\right]=I\sigma_{e}^{2}$, where A is the additive genetic relationship matrix between individuals based on a maximum of 11 generations of pedigree, $\sigma_{A}^{2}$ is the genetic variance, I an identity matrix, and $\sigma_{e}^{2}$ is the residual variance.

## Results and discussion

### Relationship between walking ability and body weight

The mean BW of the broilers differed between the GS groups. Birds with GS1 had the lowest mean BW (1,911 (SD 168) g; *n* = 153), followed by GS2 birds (1,982 (SD 154) g; *n* = 344) and subsequently GS3 birds (2,077 (SD 144) g; *n* = 87). Birds with GS4 had a mean BW of 2,005 g (SD 283; *n* = 11), which did not statistically differ from the other GS groups. This lack of a difference for GS4 is likely due to the very limited number of birds in this GS class. The observed association between BW and GS – with higher BW being associated with worse GS – is in line with reports from other studies (e.g. Riber et al., 2021).

### Relationship between top-view walking features and gait scores

Statistical analyses revealed that LBO, MEAN_SPEED, MAX_SPEED and MAX_ACC differed between GS groups, and for STEP an interaction between BW and GS was observed (Table 1). These results for the relationships between each of the different walking features and GS are discussed below. Besides differences between GS groups, differences between the walkways were observed for STEP (*p* < 0.001) and MEAN_SPEED (*p* = 0.006). This is likely related to differences between the two gait scorers in how quickly they decided to nudge the birds, as unfortunately no fixed protocol was used for the nudging.

**Table 1:** **Least squares means (SE) of the walking features per gait score group**^1^**and genetic parameters (SE) of the walking features.** In case of a statistically significant interaction with body weight, estimates are shown for three body weights (based on the mean body weight plus or minus one SD). GS = gait score group; $\sigma_{A}^{2}$ = genetic variance; $\sigma_{P}^{2}$ = phenotypic variance; $h^{2}$ = heritability; LBO = lateral body oscillation; STEP = number of steps taken; MEAN_SPEED = mean overall speed of movement; MAX_SPEED = maximum forward moving speed; MAX_ACC = maximum forward acceleration; BW = body weight; px = pixels; NE = not estimable.

**Walking feature**	**Gait score group**					**Genetic parameters**		
	GS1	GS2	GS3	GS4	p-value	$\sigma_{A}^{2}$	$\sigma_{P}^{2}$	$h^{2}$
LBO (px)	12.6 (0.23)^b^	13.7 (0.15)^a^	12.2 (0.30)^bc^	10.2 (0.82)^c^	*p* < 0.001	0.77 (0.56)	7.84 (0.46)	0.10 (0.07)
STEP (number) in interaction with BW					*p* = 0.007	5.31 (3.23)	36.16 (2.18)	0.15 (0.09)
Mean BW – 1SD (1,811 g)	16.4 (0.43)^d^	20.7 (0.37)^c^	25.5 (1.07)^b^	32.6 (2.01)^a^				
Mean BW (1,978 g)	15.7 (0.40)^d^	20.4 (0.25)^c^	27.2 (0.61)^b^	32.7 (1.72)^a^				
Mean BW + 1SD (2,145 g)	14.9 (0.65)^c^	20.1 (0.36)^b^	28.9 (0.58)^a^	32.9 (1.96)^a^				
MEAN_SPEED (px/second)	221.5 (5.29)^a^	153.6 (3.36)^b^	70.7 (6.84)^c^	53.7 (18.71)^c^	*p* < 0.001	705.13 (464.54)	6111.20 (362.27)	0.12 (0.07)
MAX_SPEED (px/second)	606 (12.72)^a^	505 (8.13)^b^	380 (16.46)^c^	416 (45.04)^bc^	*p* < 0.001	229.07 (1423.10)	26740.00 (1566.50)	0.01 (0.05)
MAX_ACC (px/second^2^)	3517 (71.0)^a^	3319 (45.4)^ab^	3100 (94.6)^b^	3254 (250.7)^ab^	*p* = 0.006	NE	NE	NE

1 Different superscript letters within a row indicate significantly different values (*p* < 0.05).

***Number of steps taken.*** Within the different BW levels, higher GS was associated with more steps taken (Table 1). This is in line with our earlier observations for crossbred broilers (Fodor et al., 2024), and with observations made by Aydin (2017). Possibly, birds with poor GS may not lift their legs as high as birds with good GS (as for example observed by Caplen et al. (2012) and Fodor et al. (2023)), and therefore cannot cover large distances per step taken.

***Lateral body oscillation.*** The highest level of LBO was observed in GS2 birds, and this was significantly higher than for all other GS groups. The LBO of GS1 birds was furthermore higher than for GS4 birds (Table 1). Some other studies – but not all, see e.g. Aydin (2017) – have also shown lower levels of LBO for birds with worse gait. For example, Li et al. (2023) observed less LBO for GS2 birds (on a three point scale) than for GS0 or GS1 birds. They suggest that this may have been due to GS2 birds sitting down or waddling instead of walking upright in their test. In our previous study (Fodor et al., 2024), we observed a tendency for a higher level of LBO in GS2 birds than in GS1 birds but no difference with GS3+ birds (although numerically the LBO for GS3+ birds was higher). It appears that recording LBO currently does not provide a clear and distinctive insight into GS.

***Mean speed of movement.*** The mean speed of movement decreased as the GS of the broilers increased from GS1 to GS3, and there was a numerical, but not statistically significant, lower mean movement speed for GS4 than GS3 birds (Table 1). This decrease in movement speed with increasing GS has also been reported by others (e.g. Li et al. (2023) and Aydin (2017)). The mean speed of movement may, however, be affected by birds resting in the walkway in between walking bouts (i.e., mean speed was defined here as the length of the track - which could differ per bird - divided by the time span used to cover that distance). It is important to note that birds were gently nudged here if they did not voluntarily walk towards the end of the walkway, although no fixed protocol was used for this and it was not noted down when birds were nudged. This nudging may have increased the mean speed – or made the mean speeds of birds in the different GS classes more similar – compared to solely voluntary walking.

***Maximum speed of movement.*** To limit the impact of resting bouts in between walking, we furthermore examined the MAX_SPEED, for which the outcomes were similar to the mean speed of movement: the maximum speed decreased with increasing GS, from GS1 to GS3, and the maximum speed of GS4 broilers was lower than for GS1 broilers but did not differ from birds with GS2 or GS3 (Table 1). This is in line with observations made by Li et al. (2023). In addition to a GS effect, a BW effect was observed for MAX_SPEED (*p* = 0.022), with a higher maximum forward moving speed for birds with higher BW. One potential explanation could be that heavier birds may also be larger birds and therefore be able to take larger steps and cover a given distance faster. When looking at the number of steps taken in this study, there was indeed a numerical decrease in number of steps taken (and thus an increase in step length) with increasing BW within GS1 and GS2, but not in GS3 and GS4, in which the opposite was observed (Table 1). One hypothesis is that the nudging of the birds that were more reluctant to walk (possibly because of their worse GS) may have impacted this. However, more research is required to provide insight into the relationship between BW and MAX_SPEED.

***Maximum forward moving acceleration.*** The MAX_ACC was less distinctive between GS classes, as only a difference between GS1 and GS3 birds was observed, with a higher maximum acceleration for GS1 than for GS3 birds (Table 1). Li et al. (2023) also recorded linear moving acceleration, but observed no differences between GS classes in maximum linear moving acceleration. However, they did observe differences between their three GS classes in average linear moving acceleration, with decreasing acceleration with increasing GS score.

### Combining the top-view walking features

**Fig. 1:**
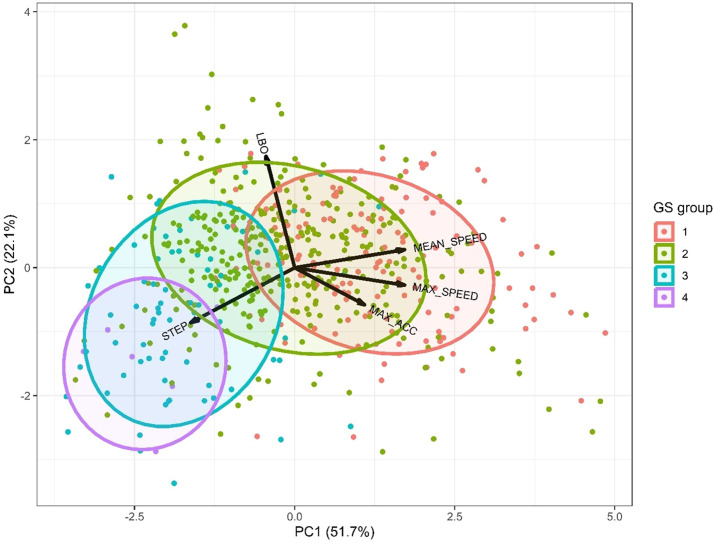
**Visual representation of the GS groups across the first two principal components of the walking features.** Different colour dots indicate individual birds with different gait scores. The coloured ellipses indicate the normal probability of the GS classes. The variables (LBO, STEP, MEAN_SPEED, MAX_SPEED and MAX_ACC) and their directions are indicated with arrows.

Fig. 1 shows a visual representation of the GS groups across the first two principal components of the walking features. Even though there is substantial overlap between the GS groups, birds with worse GS appear to show lower mean and maximum speeds and lower maximum accelerations, while showing more steps taken and lower LBO levels. This suggests that the combination of the walking features presented here can provide some insight into walking ability, although it remains challenging to assign the correct GS to birds based on these features.

### Genetic parameters

Manually determined GS and BW were found to be moderately heritable ($h^{2}$ = 0.25 ± 0.10 and $h^{2}$ = 0.40 ± 0.12, respectively). Furthermore, LBO, STEP and MEAN_SPEED were observed to have a low heritability ($h^{2}$ = 0.10 ± 0.07, $h^{2}$ = 0.15 ± 0.09 and $h^{2}$ = 0.12 ± 0.07, respectively; Table 1). MAX_SPEED showed a very low $h^{2}$ (0.01 ± 0.05) that did not differ from zero, and the models for MAX_ACC did not converge (Table 1). Kapell et al. (2025) also assessed the heritability of manually determined GS, albeit using a different scoring system (8-class scale), and observed heritabilities between 0.14 and 0.24, depending on which pure-bred commercial broiler line was assessed. This aligns well with the heritability estimate ($h^{2}$ = 0.25) for the pure-bred broiler line that was studied here. The heritability estimate for BW observed here ($h^{2}$ = 0.40) is also in line with reports in literature (e.g. Mebratie et al., 2017).

### Perspectives for implementation

The walking features LBO, STEP, and MEAN_SPEED appear suitable for inclusion in breeding programs, as they were heritable and related to walking ability. Compared with manual GS, they offer two advantages: they can be recorded automatically without trained observers, and they are continuous traits that avoid forcing inherently continuous variation into discrete classes. However, their heritabilities were low, and further work is needed to evaluate their relationships with other health, welfare, and production traits to avoid unintended negative effects of selection. Future research should also examine combinations of walking features to assess their combined potential for improving walking ability in broilers.

## Funding

The study was supported by the Dutch Ministry of Economic Affairs (TKI Agri & Food project LWV21030) and the partners Cobb, Dorset Identification B.V. and FarmResult.

## Declarations of interest

Cobb was involved in the study design and in the collection of data. The funders had no role in the decision to submit the article for publication.

## CRediT authorship contribution statement

**Malou van der Sluis:** Writing – review & editing, Writing – original draft, Visualization, Methodology, Investigation, Formal analysis, Conceptualization. **Marjaneh Taghavi:** Writing – review & editing, Writing – original draft, Visualization, Validation, Software, Methodology, Formal analysis, Conceptualization. **István Fodor:** Writing – review & editing, Writing – original draft, Visualization, Methodology, Investigation, Formal analysis, Conceptualization. **Elianne G.T. van der Valk:** Writing – review & editing, Investigation. **Jesus Arango:** Writing – review & editing, Resources. **Esther D. Ellen:** Writing – review & editing, Writing – original draft, Project administration, Methodology, Funding acquisition, Formal analysis, Conceptualization.

## Disclosures

The authors declare that they have no known competing financial interests or personal relationships that could have appeared to influence the work reported in this paper.
